# Comparison of Physical Activity Level From Osteoporosis, Parkinson and Healthy Subjects

**DOI:** 10.1093/geroni/igab046.3373

**Published:** 2021-12-17

**Authors:** Seong Hyun Moon, Thurmon Lockhart, Krupa Doshi

**Affiliations:** 1 Arizona State University, Tempe, Arizona, United States; 2 MAYO Clinic, Scottsdale, Arizona, United States

## Abstract

Lifestyle at the habitation immensely affects the progression of various illnesses, such as Osteoporosis and Parkinson’s disease (PD). These disorders lead patients to a sedentary lifestyle and result in significantly less movement compared to the average healthy individual. The combination of these backgrounds escalates the percentage of fall incidents. Quantifying physical activity levels from longitudinal Activities of Daily Living (ADL) data of these disease patients could stipulate intuition of their fall mechanisms. The objective of this study is to compare the osteoporosis, Parkinson's disease, and healthy group’s physical activity level from their ADL. For this study total of eighteen subjects participated (healthy=6, osteoporosis=6, PD=6). The result indicated that the dynamic physical activity level for the healthy subject was 13.2%, the osteoporosis subject was 7.9%, and the PD subject was 7.0%. This indicates that there was a significant decline in physical activity level for the PD compared to healthy subjects (P=0.0024*). Also, a comparison between healthy and osteoporosis subjects showed a significant difference (P=0.0066*). Lastly, the physical activity level of PD and osteoporosis subjects did not have a significant difference among them (P=0.6276). The aim of this study was to evaluate the physical activity level of the osteoporosis, PD, and healthy subjects. The systematic approach of collecting physical activity levels with the Inertial Measurement Unit (IMU) device allowed researchers to collect the quantitative data of ADL. In this experiment, healthy subjects were significantly more physically active compared to osteoporosis and PD patients.

